# Hippocampal slice preparation in rats acutely suppresses immunoreactivity of microtubule‐associated protein (Map2) and glycogen levels without affecting numbers of glia or levels of the glutamate transporter VGlut1

**DOI:** 10.1002/brb3.736

**Published:** 2017-05-30

**Authors:** Liana R. Stein, Charles F. Zorumski, Yukitoshi Izumi

**Affiliations:** ^1^ Department of Psychiatry Washington University School of Medicine St. Louis MO USA; ^2^ The Taylor Family Institute for Innovative Psychiatric Research Washington University School of Medicine St. Louis MO USA; ^3^ Center for Brain Research in Mood Disorders Washington University School of Medicine St. Louis MO USA

**Keywords:** glycogen, hippocampus, Map2, slice preparation, VGlut1

## Abstract

**Introduction:**

With its preservation of cytoarchitecture and synaptic circuitry, the hippocampal slice preparation has been a critical tool for studying the electrophysiological effects of pharmacological and genetic manipulations. To analyze the maximum number of slices or readouts per dissection, long incubation times postslice preparation are commonly used. We were interested in how slice integrity is affected by incubation postslice preparation.

**Methods:**

Hippocampal slices were prepared by three different methods: a chopper, a vibratome, and a rotary slicer. To test slice integrity, we compared glycogen levels and immunohistochemistry of selected proteins in rat hippocampal slices immediately after dissection and following 2 and 4 hr of incubation.

**Results:**

We found that immunoreactivity of the dendritic marker microtubule‐associated protein 2 (Map2) drastically decreased during this incubation period, whereas immunoreactivity of the glutamate transporter VGlut1 did not significantly change with incubation time. Astrocytic and microglial cell numbers also did not significantly change with incubation time whereas glycogen levels markedly increased during incubation.

**Conclusion:**

Immunoreactivity of the dendritic marker Map2 quickly decreased after dissection with all the slicing methods. This work highlights a need for caution when using long incubation periods following slice preparation.

## 1 INTRODUCTION

One of the most commonly studied models of mammalian brain physiology is the in vitro hippocampal slice. The hippocampal slice offers a unique opportunity to study synaptic function in a forum that preserves the architecture and circuitry of the hippocampus (Schwartzkroin, 1975). As such, it has long been a critical tool for assessing electrophysiological effects of pharmacological and genetic manipulations. Hippocampal slices are also useful for histological assessment of experimental pathology. For instance, we have reported that excitotoxic damage induced by glutamate agonists can be well characterized using hippocampal slices (Izumi, Benz, Katsuki, & Zorumski, 1997).

Synaptic transmission in hippocampal slices is known to be altered by variables present during slice preparation, such as incubation temperature, oxygenation, and dissection procedure (Villers & Ris, 2013; Watson, Weiner, & Carlen, 1997). However, the time between slice preparation and recording has received less attention and has not been standardized by the field (Sajikumar, Navakkode, & Frey, 2005). Even though maximal evoked electrical responses can be obtained within 30–60 min of incubation, it is common practice to incubate slices for 1–2 hr, regardless of temperature, glucose levels, or oxygen levels of incubation, to allow recovery from the trauma of preparation and the development of a stable metabolic state prior to beginning experiments (Kirov, Sorra, & Harris, 1999; Schurr, Reid, Tseng, & Edmonds, 1984; Wang & Kass, 1997; Whittingham, Lust, Christakis, & Passonneau, 1984). Both electrophysiologically and metabolically, this stable state has been shown to persist for up to 6–9 hr of incubation, at which point deterioration and electrical failure begin and proceed rapidly (Chang & Greenough, 1984; Schurr et al., 1984; Whittingham et al., 1984). As a result, current protocols posit that slices can be stored in oxygenated CSF for 4–6 hr before their integrity is compromised enough to result in unstable electrical recordings (Bortolotto, Amici, Anderson, Isaac, & Collingridge, 2011; Lein, Barnhart, & Pessah, 2011).

To our knowledge, only a handful of studies have investigated slice integrity as a function of time after incubation (Chang & Greenough, 1984; Fiala et al., 2003; Schurr et al., 1984). Prior studies showed that general cytoarchitecture was well‐preserved after 6 hr (Schurr et al., 1984). However, these studies have not focused on changes in synaptic and other proteins. Microtubule‐associated protein 2 (Map2), a dendritic phosphoprotein, is one of the most abundant cytoskeletal proteins and the most vulnerable upon neuronal injury, making it a sensitive and early biomarker for neuropathology (Folkerts, Berman, Muizelaar, & Rafols, 1998; Hoskison, Yanagawa, Obata, & Shuttleworth, 2007; Matesic & Lin, 1994). We hypothesized that Map2 may be altered in the early phase of incubation. Because glutamate excitotoxicity is one major cause of neuronal dysfunction upon injury (Aarts & Tymianski, 2004); we also hypothesized that incubation period would positively correlate with changes in glutamate release machinery. Vesicular glutamate transporters (VGluts) are presynaptic proteins located on synaptic vesicles that translocate glutamate into the synaptic vesicle lumen, protecting it from degradation before calcium‐dependent, exocytotic release into the synaptic cleft (Santos, Li, & Voglmaier, 2009). Because VGlut1 levels determine the amount of glutamate stored and released per vesicle, and variations in VGlut1 expression regulate the efficacy of glutamate synaptic transmission (Santos et al., 2009), we examined VGlu1 immunohistochemistry. Furthermore, because astrocytes and microglia respond to injury by increasing their pool and cell size (Nikonenko, Radenovic, Andjus, & Skibo, 2009), we also hypothesized that the period of slice incubation may progressively induce gliosis and examined Gfap and Ibal as glial markers.

A secondary consideration is that prior studies typically prepared slices by a tissue chopper, which is less commonly used in modern neuroscience. Since the method of tissue preparation affected metabolite uptake and preservation (de Barry, Langley, Vincendon, & Gombos, 1982; Garthwaite, Woodhams, Collins, & Balazs, 1979), the method of tissue preparation may differentially affect slice integrity during incubation. Thus, in this study, we prepared hippocampal slices with three different slicers: a tissue chopper, a vibratome, and a rotary slicer.

## 2 MATERIALS AND METHODS

### 2.1 Animals

All animal procedures were approved by the Washington University Animal Studies Committee, Division of Comparative Medicine, Washington University School of Medicine, St. Louis, MO, and were in accordance with the National Institute of Health Guide for the Care and Use of Laboratory Animals (NIH Publications No. 80‐23). All efforts were made to minimize the number of animals used and their suffering.

### 2.2 Hippocampal slice preparation

Hippocampal slices were prepared from postnatal day 33–35 Sprague–Dawley rats purchased from Harlan (Indianapolis, IN) using standard methods (Stein & Imai, 2014) with a rotary slicer, a vibratome, or a tissue chopper. Rats were moved to the dissection room the night prior to sacrifice and dissections were performed between 10 a.m. and 12 p.m. to account for possible effects of transportation and the light/dark cycle, respectively. Briefly, rats were anesthetized with isoflurane and decapitated. Hippocampi were rapidly dissected and placed in 4–6°C artificial cerebrospinal fluid (ACSF) containing (in mM): 124 NaCl, 5 KCl, 2 MgSO_4_, 2 CaCl_2_, 1.25 NaH_2_PO_4_, 22 NaHCO_3_, 10 glucose, gassed with 95% O_2_–5% CO_2_ and sectioned transversely into 500 μm slices. This thickness was chosen to allow comparisons with previous protocols (Whittingham et al., 1984). Because slicing hippocampi at 4°C rather than 37°C does not affect metabolic profiles and electrical responses (Whittingham et al., 1984), sectioning by a vibratome and rotary slicer was done in 4–6°C ACSF. The chopper and vibratome were used according to standard protocols (Wang & Kass, 1997). In all three slicing techniques, the dentate gyrus was placed face down at the time slices were cut. After slicing, the tissue sections were placed in a 4–6°C ACSF bath for 1, 2, or 4 hr of incubation as specified. This bath was bubbled with oxygen at a flow rate of 2 ml/min and the temperature was closely monitored by a YSI model 73ATA indicating controller. To eliminate possible bias caused by changes in solution pH, dissolved oxygen, and temperature, the same number of animals were subjected to each technique on each dissection day. All slices were from the dorsal hippocampus.

For the tissue chopper (Brinkmann Instruments), we briefly submerged hippocampi in chilled ACSF then placed it on a filter paper perpendicular to the blade and finished slicing in less than 1 min. The chopped hippocampal slices were immediately transferred to ACSF. For the vibratome (Vibroslice, SYS‐NYSLM1), we mechanically stabilized hippocampi by pinning them vertically in a channel carved into a 3% round agar block placed in a bath of ACSF. For the rotary slicer (made by Prof. Hiroshi Kato, Yamagata University), we pinned hippocampi horizontally in a channel carved into a 3% round agar block placed in a bath of ACSF. For immunohistochemistry and glycogen analyses, slices were immediately used (0 time point) or incubated in gassed ACSF for 2 or 4 hr at 30°C.

### 2.3 Immunohistochemistry

Hippocampal slices were fixed in phosphate‐buffered solution of 4% paraformaldehyde (PFA) overnight, equilibrated in 15% sucrose overnight, then equilibrated in 30% sucrose overnight, frozen, and stored at −80°C until sectioning. The first and last 100 μm of the 500 μm sections were discarded. The inner 300 μm of each slice was cut into 30 μm sections in a 1 in 4 series by a cryostat and stored at −30°C in cryoprotectant until use. Tissue sections were incubated with 3% H_2_O_2_ for 15 min to remove endogenous peroxidase activity. Tissue sections were incubated in blocking/permeabilization solution containing 10% normal goat serum, 1% BSA, and 0.3% Triton‐X in PBS for 45–60 min prior to 24 or 48 hr of incubation with primary antibodies in 5% normal goat serum and 0.1% Triton‐X in PBS at 4°C at the following concentrations: Gfap (1:1,000; Millipore, MAB360, mouse, RRID:AB_2109815), Iba1 (1:500; Wako, #019‐19741, rabbit), Map2 (1:500, Sigma, M9942, mouse, RRID:AB_477256), VGlut1 (1:1,000, Synaptic Systems, 135 304, guinea pig, RRID:AB_887878). Antibody specificity was determined by lack of staining after omission of primary or secondary antibodies and upon replacing the primary antibody with a species‐specific IgG control. Alexa647 (1:200), Alexa488 (1:200), or Cy3 (1:400) conjugated‐secondary antibodies (Jackson ImmunoResearch) diluted in 2% normal goat serum, 1% BSA, and 0.1% Triton‐X in PBS were added for 2 hr at room temperature. Detection of Map2 was performed using the TSA‐Plus kit (PerkinElmer, Boston, MA). Nuclei were stained with 4,6‐diamidino‐2‐phenylindole (Sigma) for 10 min at room temperature. High‐magnification (20x, 0.8DICII) microscopic imaging was performed using a Zeiss Axioimager.Z1 (Map2, VGlut1, Gfap, Iba1) or an Olympus NanoZoomer 2.0‐HT (H&E). Map2 and Iba1 were detected using the Cy3 filter. VGlut1 and Gfap were detected using the GFP filter. Images were taken in z‐stacks of 1 μm steps through the range of tissue section immunoreactivity. ImageJ was used to 3D render z‐stacks. All quantification was performed in 4 rats per condition, 2–4 slices per rat, and 1 field of view per slice. Quantification of Map2 and VGlut1 immunoreactivity was performed by gray value measurements using ImageJ of the denoted regions. Background immunoreactivity was determined by taking a gray value measurement of a nonimmunoreactive area of each individual image and subtracted. Numbers of Gfap+ and Iba1 +  cells were quantified by cell counting and normalized by the area of tissue quantified.

### 2.4 Glycogen measurements

Because brain glycogen has a circadian rhythm (Hutchins & Rogers, 1970), we performed all dissections between 10 a.m. and 12 p.m. each day. Hippocampal slices were flash frozen in liquid nitrogen, and stored at −80°C until use, a protocol which preserves brain glycogen levels (Hutchins & Rogers, 1970). To measure glycogen, hippocampal slices were homogenized with a 25G syringe in 100 μl of water on ice immediately upon thawing. Homogenates were boiled for 5 min to inactivate enzymes and centrifuged at 13,000 × *g* for 5 min to remove insoluble material. Glycogen was measured using a Glycogen Assay Kit (Sigma, MAK016), according to manufacturer's instructions. The value obtained in each sample was normalized by the protein concentration in that sample. This protein concentration was determined by a Bradford assay.

### 2.5 Statistical analyses

Numerical data are presented as mean ± standard error of the mean (*SEM*). Statistical significance was determined by Analysis of variance (ANOVA). Statistical analyses were performed using R (RRID:SCR_001905) or SigmaPlot 5.01 and 9.0 (RRID:SCR_003210) and SigmaStat 3.1 (Systat Software Inc., Richmond, CA, RRID:SCR_010285). *p*‐Values of less than .050 were considered significant. Sample sizes are stated in figure legends and refer to individual rats. Statistical analyses of the immunohistochemical data are summarized in Table 1.

**Table 1:** Statistical analyses of the immunohistochemical data

Figure panel	Immunostain	Statistical method	Comparison	Significance
2B	Map2	2‐way ANOVA	Incubation time	*F*_(2,64)_=5.99, *p* = .004
			Slicing method	*F*_(2,64)_=2.08, *p* = .13
2C	Map2	2‐way ANOVA	Incubation time	*F*_(2,64)_=12.57, *p* = .00002
			Slicing method	*F*_(2,64)_=4.33, *p* = .02
3B	VGlut1	2‐way ANOVA	Incubation time	*F*_(2,64)_=1.95, *p* = .15
			Slicing method	*F*_(2,64)_=5.85, *p* = .005
3C	VGlut1	2‐way ANOVA	Incubation time	*F*_(2,64)_=0.513, *p* = .601
			Slicing method	*F*_(2,64)_=4.51, *p* = .015
4B	Gfap	2‐way ANOVA	Incubation time	*F*_(2,31)_=1.65, *p* = .21
			Slicing method	*F*_(2,31)_=1.23, *p* = .31
4C	Iba1	2‐way ANOVA	Incubation time	*F*_(2,31)_=1.26, *p* = .23
			Slicing method	*F*_(2,31)_=4.72, *p* = .016

## 3 RESULTS

### 3.1 Glycogen levels increase during slice incubation regardless of slicing method

**Figure 1:**
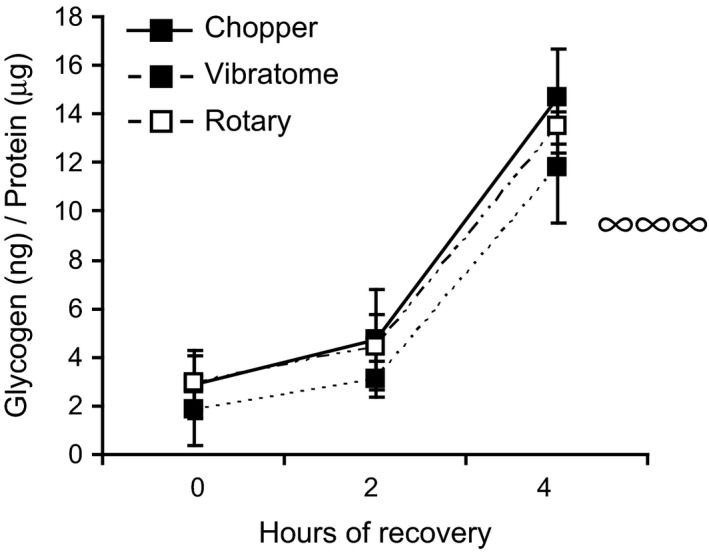
Incubation period strongly affects glycogen levels and tissue morphology of hippocampal slices. Glycogen levels in hippocampal slices generated by the tissue chopper, vibratome, and rotary slicer (*n* = 3–8) after 0, 2, and 4 hr of recovery in ACSF. Data are presented as mean ± *SEM*. ∞ represents a significant Time effect generated by ANOVA. ^∞∞∞^ *p* < .001

Hippocampal slice preparation is known to alter the levels of multiple metabolites acutely (Whittingham et al., 1984). One critical neural metabolite is glycogen. Astrocytic glycogen breakdown and monocarboxylate release are essential for long‐term memory formation and for the maintenance of long‐term potentiation (Suzuki et al., 2011). Glycogen turns over rapidly in the brain and this turnover is enhanced when neural activity is increased: 6–50‐fold during physiological stimulation and more than 200‐fold during energy deprivation (Dienel & Cruz, 2006). After dissection, glycogen levels fall precipitously, but transiently (Fiala et al., 2003; Hutchins & Rogers, 1970; Lipton, 1989; McIlwain & Tresize, 1956; Wender et al., 2000). To verify that our slices are metabolically similar to previous reports, we assessed glycogen levels immediately postslice preparation, and 2 and 4 hr postslice preparation. As reported, glycogen levels were depleted immediately postdissection, but significantly increased with time (ANOVA, [*F*_(2,45)_=42.1, *p* < .0001], TukeyHSD post‐hoc). Post‐hoc tests revealed significant differences between 0 and 2 hr (*p* < .0001) as well as 0 and 4 hr time points (*p* < .0001). Specifically, glycogen increased 1.5–1.7‐fold 2 hr after dissection and 4.6–6.4‐fold 4 hr later (Figure 1), as previously reported (Lipton, 1989). To our knowledge, a comparison of glycogen recovery among slicing methods has not been investigated. However, we did not detect a difference in either the time course of glycogen level changes or absolute levels of glycogen among slicing protocols. Thus, while glycogen is low immediately after hippocampal slice preparation and increases with time, these levels are not differentially altered by slicing method.

### 3.2 Immunohistochemical Map2 immunoreactivity is rapidly affected by slice incubation

**Figure 2:**
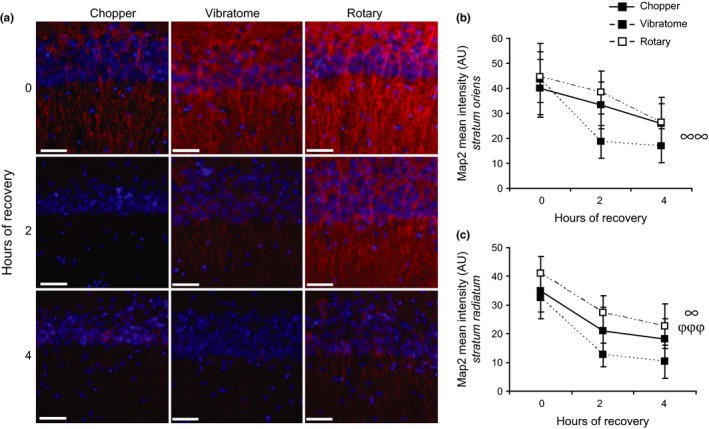
Incubation period strongly affects Map2 immunoreactivity in hippocampal slices. (a) Dendritic (Map2, red) and Dapi (blue) immunostaining of hippocampal slices generated by the tissue chopper, vibratome, and rotary slicer after 0, 2, and 4 hr of recovery in ACSF. (b) Quantification of the mean intensity of Map2 immunostaining in the *striatum oriens*. (a, b) Scale bars represent 50 μm. **C,** Quantification of the mean intensity of Map2 immunostaining in the *striatum radiatum*. (b, c) The notation of **“**0, 2, 4” on the x‐axes stand for hours of recovery postslice preparation in ACSF. Data are presented as mean ± *SEM*. ∞ represents a significant time effect generated by ANOVA. φ represents a significant method effect generated by ANOVA. ^∞^ *p* < .05. ^∞∞,φφ^ *p* <.01

Having confirmed that our slices are metabolically similar, we next examined dendritic integrity using immunohistochemistry (Figure 2a). Map2 immunoreactivity significantly declined with time in both *stratum oriens* (Figure 2b, ANOVA, [*F*_(2,64)_=5.99, *p* = .004]) and *stratum radiatum* of area CA1 (Figure 2c, ANOVA, [*F*_(2,64)_=12.57, *p* = .00002]). In *stratum oriens*, the decline in Map2 immunoreactivity with time was not statistically significant among slicing methods (ANOVA, [*F*_(2,64)_=2.08, *p* = .13]). On the other hand, the decline in Map2 immunoreactivity with time was statistically significant among slicing methods in *stratum radiatum* (ANOVA, [*F*_(2,64)_=4.33, *p* = .02]), with specific differences between the vibratome and rotary slicer (TukeyHSD post‐hoc, *p* = .01). In *stratum radiatum*, rotary‐generated slices had a nonsignificant but consistent trend towards higher levels of Map2 immunoreactivity than chopper‐generated slices, which had a consistent trend towards higher levels of Map2 immunoreactivity than vibratome‐generated slices.

**Figure 3:**
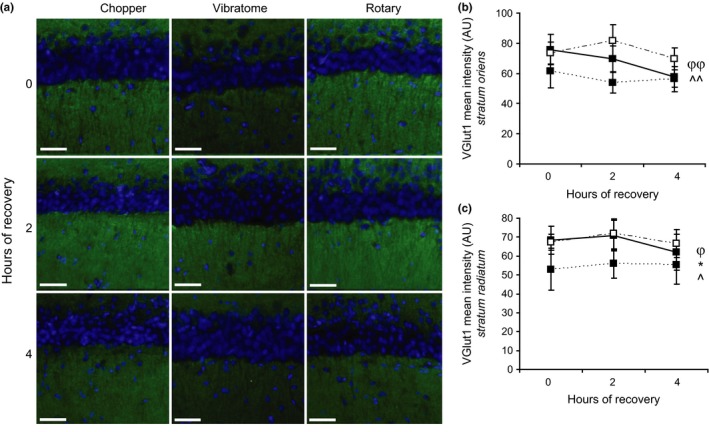
Incubation period does not affect the presynaptic glutamate marker VGlut1 in hippocampal slices. (a) VGlut1 (green) and Dapi (blue) immunostaining of hippocampal slices generated by the tissue chopper, vibratome, and rotary slicer after 0, 2, and 4 hr of recovery in ACSF. (b) Quantification of the mean intensity of VGlut1 immunostaining in the *striatum oriens*. (c) Quantification of the mean intensity of VGlut1 immunostaining in the *striatum radiatum*. (b, c) The notation of **“**0, 2, 4” on the x‐axes stand for hours of recovery postslice preparation in ACSF. Data are presented as mean ± *SEM*. ∞ represents a significant Time effect generated by ANOVA. φ represents a significant Method effect generated by ANOVA. *^,,^^ represent significance generated by the TukeyHSD post‐hoc test. * represents significance between the chopper and vibratome. ^^^ represents significance between rotary and vibratome. *^,^,φ^ *p* <.05. ^^^,φφ^ *p* <.01

Unlike Map2, immunoreactivity of VGlut1 did not change with the incubation period in either *stratum oriens* or *stratum radiatum* (Figure 3,a, b, c). However, the slicing method significantly affected VGlut1 immunoreactivity in both regions (ANOVA, *oriens*: [*F*_(2,64)_=5.85, *p* = .005]; *radiatum*: [*F*_(2,64)_=4.51, *p* = .015]). In *stratum oriens*, VGlut1 immunoreactivity was significantly lower in vibratome‐generated sections than rotary‐generated ones (TukeyHSD post‐hoc, *p* = .003). Chopper‐generated slices initially showed strong VGlut1 immunoreactivity that decreased at the 2 and 4 hr time points. In *stratum radiatum*, VGlut1 immunoreactivity was significantly lower in vibratome‐generated sections than both rotary‐ (TukeyHSD post‐hoc, *p* = .020) and chopper‐ (TukeyHSD post‐hoc, *p* = .040) generated slices. Here, chopper‐generated slices showed similarly strong VGlut1 immunoreactivity as rotary‐generated slices.

### 3.3 Slice incubation does not differentially affect astrogliosis and microgliosis

**Figure 4:**
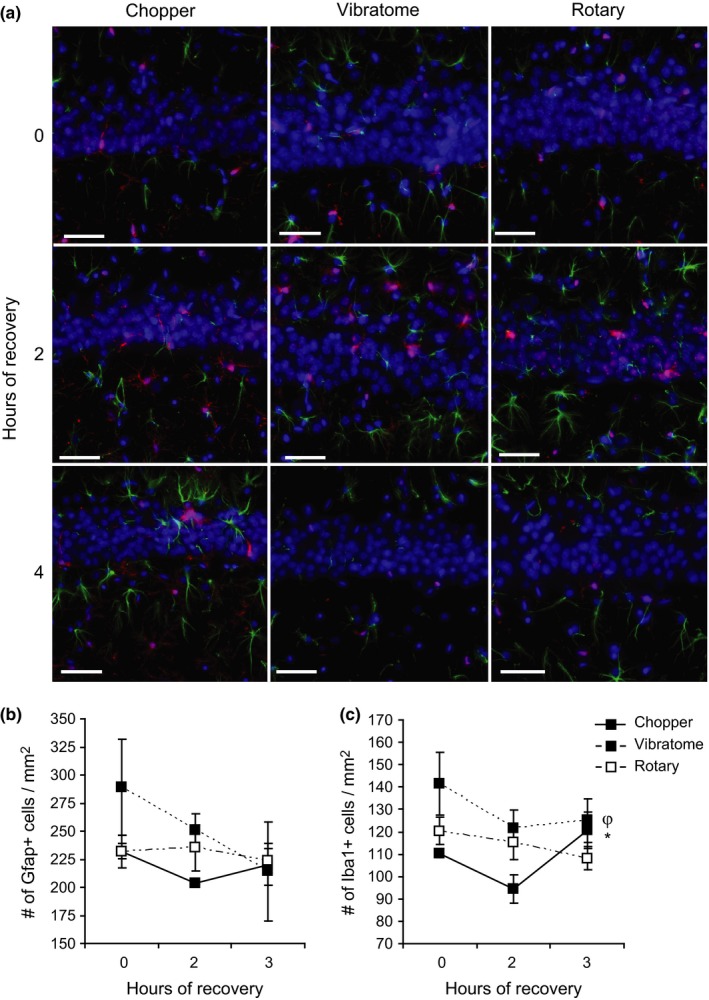
Incubation period does not affect the number of astrocytes or microglia in hippocampal slices. (a, b, c) Astrocytic (Gfap, green) and microglial (Iba1, red) immunostaining of hippocampal slices generated by the tissue chopper, vibratome, and rotary slicer after 0, 2, and 4 hr of recovery in ACSF. Scale bars represent 50 μm. Data are presented as mean ± *SEM*. ^φ^ represents a significant Method effect generated by ANOVA. * represents significance between the chopper and vibratome generated by the TukeyHSD post‐hoc test. *^,φ^ *p* < .05

Because astrocytes and microglia respond to injury by increasing their pool and cell size (Nikonenko et al., 2009), we hypothesized that the period of slice incubation would progressively induce gliosis. However, immunohistochemistry for the astrocyte marker Gfap at 0, 2, and 4 hr postslicing revealed that incubation time postslice preparation did not have a significant effect on astrogliosis (Figure 4a, b; ANOVA, Gfap: [*F*_(2,31)_=1.65, *p* = .21]). Immediately after dissection and 2 hr later, vibratome‐generated slices tended to have higher glial levels, rotary‐generated slices had intermediate levels, and chopper‐generated slices had the lowest glial levels. However, this was not statistically significant (ANOVA, [*F*_(2,31)_=1.23, *p* = .31]), and slices generated by all methods exhibited similar levels of astrocytes after 4 hr of incubation. Immunohistochemistry for the microglial marker Iba1 also yielded no significant effect of incubation time postslice preparation (Figure 4a, c; ANOVA, [*F*_(2,31)_=1.26, *p* = .23]). However, Iba1 cell counts were significantly lower in chopper‐generated slices relative to vibratome‐generated slices (ANOVA, [*F*_(2,31)_=4.72, *p* = .016], TukeyHSD post‐hoc *p* = .013). In summary, hippocampal slice preparation does not induce gliosis and slicing method has mild effects on microglial number.

## 4 DISCUSSION

Here, we assessed the effect of postslicing incubation period on the metabolic and immunohistochemical characteristics of chopper‐, vibratome‐, and rotary‐generated hippocampal slices. Tissue damage on the cut edges of hippocampal slices generates metabolic changes (Whittingham et al., 1984). Since glycogen is a major, functionally relevant, brain energy reserve (Suzuki et al., 2011; Wender et al., 2000), we measured glycogen levels in hippocampal slices. We found that glycogen levels were depleted immediately after dissections but increased dramatically between dissection and 4 hr of incubation, reaching the same levels as those reported in vivo (Lipton, 1989). Our work corroborates previous work which found that glycogen content fell 52%–68% (Lipton, 1989; Wender et al., 2000) during the first hour of incubation, but almost completely recovered after 3–4 hr (Fiala et al., 2003; Lipton, 1989). A similar time course has been observed for other metabolites (Whittingham et al., 1984). To our knowledge, no study has compared glycogen content among slicing methods. Even though we confirmed that glycogen levels recover postdissection, we did not detect a difference in glycogen levels among slicing methods. This lack of difference in glycogen levels is interesting with regards to the other metabolites that are differentially affected by slicing method, such as cyclic GMP levels (Garthwaite et al., 1979) and amino acid uptake (de Barry et al., 1982).

The dramatic decline in immunoreactivity of the dendritic marker, Map2, by 4 hr of incubation was unexpected. Even though declines in dendritic Map2 expression are relatively common, being observed in rodents and/or humans upon NMDA exposure (Hoskison et al., 2007), oxygen‐glucose deprivation (Buddle et al., 2003), anoxia (Kwei, Jiang, & Haddad, 1993), ischemia (Kitagawa et al., 1989; Kuhn, Meissner, & Oehmichen, 2005), and traumatic brain injury (Folkerts et al., 1998), it was surprising to see a strong loss of Map2 immunoreactivity so early postslice preparation. Indeed, previous work had estimated hippocampal slice lifetime to be 12 ± 4 hr, with eventual deterioration and loss of viability by 24 hr (Lynch & Schubert, 1980; Schurr et al., 1984; Wang & Kass, 1997). Viability and electrical waveform parameters are stable up to 8 hr after slicing (Leonard, Barnes, Rao, Heissenbuttel, & McNaughton, 1991), after which amplitude latency gradually declines (Schurr et al., 1984). While the rapidity in decline in Map2 immunoreactivity that we saw at 2 hr postslice preparation was unexpected, it is chronologically consistent with losses in Map2 immunoreactivity resulting from other pathophysiological events (Buddle et al., 2003; Kitagawa et al., 1989; Kwei et al., 1993; Matesic & Lin, 1994; Pettigrew et al., 1996). Our findings suggest that extreme care should be used in evaluating slices incubated for long periods after dissection, and that it may be beneficial to limit the amount of time that a single brain slice is used for recording. While all three slicing methods that we tested produced these same changes in Map2 expression, it is possible that incubation method is a significant variable in this regard. Indeed, we used bath, submerged incubation, but did not test incubating the slices in an interface chamber. It will be interesting for future work to assess the effect of incubation method on Map2 expression. It will also be important to test the expression of other markers of microtubule integrity, such as tubulin and tau. Indeed, we used one Map2 antibody (clone HM−2, Sigma), and it is possible that this antibody is sensitive to a particular posttranslational modification, resulting in changes in immunoreactivity that do not reflect changes in protein levels.

To our knowledge, only three prior studies have investigated hippocampal histology postslicing. One showed that mitochondrial area, bouton area, synapse number, spine number, and spine head perimeter did not change between 15 min, 2 hr, and 8 hr of incubation (Chang & Greenough, 1984). Another found that general cytoarchitecture was well‐preserved after 6 hr, but deteriorated by 12 hr (Schurr et al., 1984). After 12 hr of incubation, the majority of the pyramidal cell nuclei were pyknotic with electron‐opaque cytoplasms containing scattered microtubular bundles, vacuoles, and burst mitochondria and neuropil characterized by many darkly stained neurites mingled with swollen dendrites (Schurr et al., 1984). The third study demonstrated that 3 hr after slicing, there were no changes in synaptogenesis, synapse volume density, or dendritic spine density. Moreover, dendritic microtubule length and coated vesicle number had completely recovered (Fiala et al., 2003). Putting these findings together with ours suggests that loss of Map2 immunoreactivity is one of the first detectable changes and precedes organelle damage. Combining this finding with ours could indicate that synaptic deterioration may be occurring between 3 and 4 hr of incubation. The finding that immunohistochemical readouts are more sensitive than electrophysiological readouts may explain previous reports of robust electrophysiological response. For example, others have found that hippocampal fEPSPs could be evoked in slices prepared under suboptimal conditions, such as 3 hr after death (Leonard et al., 1991). Our work suggests that tissue integrity may not have been maintained in this context.

Mechanistically, we are still seeking to understand why Map2 immunoreactivity is lost so rapidly after hippocampal slice preparation. This rapid loss likely occurs for the same reason that Map2 immunoreactivity promptly disappears after ischemia (Kitagawa et al., 1989); a phenomenon which is also not understood. One potential mediator is calcium‐dependent phosphorylation. Map2 loss in response to NMDA is calcium‐dependent (Hoskison et al., 2007) and injury‐related increases in intracellular calcium concentrations can alter kinase activity (Folkerts et al., 1998). Since Map2 phosphorylation is necessary for the binding of Map2 to microtubules and other proteins, altered kinase activity could thus affect Map2 assembly (Folkerts et al., 1998). Candidate kinases known to phosphorylate Map2 include cyclic AMP‐dependent kinase (PKA), calcium/calmodulin‐dependent kinase, protein kinase C, and Map2 kinase (Sanchez, Diaz‐Nido, & Avila, 2000). Another potential mediator is calpain, which degrades the neuronal cytoskeleton, preferentially targeting Map2, very early after ischemia. Indeed, in ischemic tissue, an increase in calpain activity paralleled loss of dendritic Map2 +  immunoreactivity (Nikonenko et al., 2009; Pettigrew et al., 1996).

It is intriguing that glycogen levels recovered postslice preparation during the same time window that Map2 immunoreactivity declined. One reason for these converse findings may be cell type specificity. Although Map2 and VGlut1 immunoreactivity resides in neurons, glycogen is exclusively localized in and metabolized by astrocytes to provide physiologically significant levels of energy upon breakdown to lactate, which is transferred to neurons for fuel (Dienel & Cruz, 2006; Wender et al., 2000).

The decline in Map2 +  occurred in the absence of changes in levels of the glutamate transporter VGlut1, numbers of astrocytes, or numbers of microglia. Because other forms of insult and injury increased VGlut1 immunoreactivity, such as acutely after ischemic insult (Sanchez‐Mendoza et al., 2010), kainate injection (Lobo et al., 2011), and chronic stress (Farley, Dumas, El Mestikawy, & Giros, 2012), we expected VGlut1 immunoreactivity to increase after slice preparation. In contrast, we found that VGlut1 immunoreactivity significantly decreased postslice preparation in chopper‐generated slices, but stayed relatively constant in slices generated by a vibratome or rotary slicer. One potential reason for the decline in VGlut1 immunoreactivity after slice preparation is glutamate excitotoxicity (Lobo et al., 2011; Melo et al., 2013). While we did not observe a change in astrocyte or microglial cell number, it remains possible that these cell types underwent pathological changes in morphology.

In conclusion, we tested the impact of incubation period on hippocampal slices prepared by three different methods and found that immunoreactivity of the dendritic marker Map2 quickly decreased after dissection with all the slicing methods. The findings are important because most protocols recommend more than 1 hr of recovery followed by several hours of recordings (Bortolotto et al., 2011; Lein et al., 2011). Thus, this finding suggests the need for caution in using hippocampal slices long after their preparation even if cytoarchitecture and synaptic responses are preserved.

## DISCLOSURES

C.F.Z. serves on the scientific advisory board of Sage Therapeutics. L.R.S. and Y.I. declare no competing financial interests.

## AUTHOR CONTRIBUTION

L.R.S., Y.I., and C.F.Z. designed research, analyzed data, and wrote the manuscript. L.R.S. and Y.I. performed research.
